# The Medical Education Partnership Initiative (MEPI): Innovations and Lessons for Health Professions Training and Research in Africa

**DOI:** 10.29024/aogh.8

**Published:** 2018-04-30

**Authors:** Francis Omaswa, Elsie Kiguli-Malwadde, Peter Donkor, James Hakim, Miliard Derbew, Sarah Baird, Seble Frehywot, Onesmus Wairumbi Gachuno, Steve Kamiza, Isaac Ongubo Kibwage, Alfred Mteta Kien, Yakub Mulla, Fitzhugh Mullan, Jean B. Nachega, Oathokwa Nkomazana, Emilia Noormohamed, Vincent Ojoome, David Olalaye, Sandy Pillay, Nelson K. Sewankambo, Marietjie De Villiers

**Affiliations:** 1 African Centre for Global Health and Social Transformation/Uganda, African Centre for Global Health and Social Transformation, 13B Acacia Avenue, Kampala, UG; 2 Kwame Nkrumah University of Science and Technology/Ghana, Accra Road, Kumasi, GH; 3 University of Zimbabwe/Zimbabwe, University of Zimbabwe Campus, Harare 00263, ZW; 4 Addis Ababa University/Ethiopia, King George VI St, Addis Ababa 1000, Ethiopia, Addis Ababa, ET; 5 George Washington University/USA 950 New Hampshire Ave NW Washington, DC 20052, US; 6 University of Nairobi/Kenya, GPO, Nairobi, KE; 7 University of Malawi/Malawi, College of Malawi, Private Bag 360, Chichiri, Blantyre 3, MW; 8 Kilimanjaro Christian Medical University College/Tanzania, Moshi Tanzania, TZ; 9 University of Zambia/Zambia, Great East Road, Lusaka, ZM; 10 Stellenbosch University/South Africa, Stellenbosch, 7599, ZA; 11 University of Botswana/Botswana, 4775 Notwane Road, Private Bag UB0022 Gaberone, BW; 12 Universidade Eduardo Mondlane/Mozambique, Avenida do Zimbabwe, Maputo, MZ; 13 University of Ibadan/Nigeria, UI Road, Ibadan, Oyo State, NG; 14 University of KwaZulu-Natal/South Africa, King George V Ave, Durban, 4041, ZA; 15 Makerere University/Uganda, Kampala, UG

## Abstract

MEPI was a $130 million competitively awarded grant by President’s Emergency Plan for AIDS Relief (PEPFAR) and National Institutes of Health (NIH) to 13 Medical Schools in 12 Sub-Saharan African countries and a Coordinating Centre (CC). Implementation was led by Principal investigators (PIs) from the grantee institutions supported by Health Resources and Services Administration (HRSA), NIH and the CC from September, 2010 to August, 2015. The goals were to increase the capacity of the awardees to produce more and better doctors, strengthen locally relevant research, promote retention of the graduates within their countries and ensure sustainability. MEPI ignited excitement and stimulated a broad range of improvements in the grantee schools and countries. Through in-country consortium arrangements African PIs expanded the programme from the 13 grantees to over 60 medical schools in Africa, creating vibrant South–South and South–North partnerships in medical education, and research. Grantees revised curricular to competency based models, created medical education units to upgrade the quality of education and established research support centres to promote institutional and collaborative research. MEPI stimulated the establishment of ten new schools, doubling of the students’ intake, in some schools, a three-fold increase in post graduate student numbers, and faculty expansion and retention.

Sustainability of the MEPI innovations was assured by enlisting the support of universities and ministries of education and health in the countries thus enabling integration of the new programs into the regular national budgets. The vibrant MEPI annual symposia are now the largest medical education events in Africa attracting global participation. These symposia and innovations will be carried forward by the successor of MEPI, the African Forum for Research and Education in Health (AFREhealth). AFREhealth promises to be more inclusive and transformative bringing together other health professionals including nurses, pharmacists, and dentists.

## Background

Africa carries 24% of the global disease burden with only 3% of the world’s Health Workforce (HWF) and less than 1% of the world’s financial resources available for health [1]. Despite significant gains, the HIV/AIDS epidemic, for example, persists in sub-Saharan Africa, costing lives, and retarding development. The Medical Education Partnership Initiative (MEPI) was funded with US$130 million from 2010 to 2015 by the President’s Emergency Plan for AIDS Relief (PEPFAR) and the National Institutes of Health (NIH) as a unique response to the African Health Workforce (HWF) crisis [2,3]. The overall goal of MEPI was to enhance models of medical education in sub-Saharan Africa. These models were intended to support PEPFAR’s goal of increasing the number of new health care workers by 140,000, strengthening medical education systems in the countries, and building clinical and research capacity in Africa [2].

WHO reported that globally, the needs-based shortage of health care workers in 2013 was estimated to be approximately 17.4 million with the most severe challenges in the African Region. The global needs-based shortage of health care workers is projected to be still above 14.5 million in 2030 (a decline of only 17%). In the African Region and in low income countries, the needs-based shortage is actually forecasted to worsen (by 45% and 33%, respectively) between 2013 and 2030 [4]. The World Health Report 2006 identified 57 countries, 36 of them in Africa, with critical shortages of health workers [1]. The sub-Saharan African Medical School Study found that the upheavals at the end of the 20th century had left most African medical schools in disrepair and demoralised, lacking essential infrastructure, depleted of faculty, and recording low levels of student enrolment [5]. PEPFAR, the NIH, and the United States Agency for International Development (USAID) also established that HWF shortages were the single most important limiting factor to the rapid scale-up of the emergency response to HIV/AIDS that was ravaging Africa. These agencies were engaged with the World Health Organization (WHO) and other partners to find and implement solutions, including the WHO Task Shifting Guidelines and education reform [6]. The Global Health Workforce Alliance (GHWA) Report on Scaling up Education and Training [7] and the Lancet Commission on Educating Health Professionals for the 21^st^ Century also proposed innovative HWF education approaches [8]. The GHWA was launched to coordinate and galvanise international action, culminating in convening of the First Global Forum on Human Resources for Health in 2008, which adopted the Kampala Declaration and Agenda for Global Action in which scaling up education and training of the HWF was a major pillar [9]. Further to this, more work has been done resulting in new political commitment to HRH, including the WHO Code on International Recruitment of Health Personnel, development of the Global Strategy on HRH 2030, the UN Commission on Health Employment and Economic Growth as well as the individual country commitments made later on [10,11,12,13].

MEPI awards were to 13 medical schools in 12 African countries with partners in the United States of America (USA) and other countries. An additional award was made to a Coordinating Centre (CC), the George Washington University (GWU) together with the Uganda-based African Centre for Global Health and Social Transformation (ACHEST). The CC task was to facilitate the collaborative work of the MEPI schools such as annual symposia, site visits, the website, and newsletters, webinars, and Technical Working Groups (TWG). The NIH and the Health Resources and Services Administration (HRSA) administered the program for the US Government. Three types of grants were awarded through a competitive peer review process, namely programmatic awards to 11 schools; five of which also received linked awards focused on non-communicable diseases and priority health areas related to or beyond HIV/AIDS, and two pilot awards targeting strengthening emergency medicine in Ghana and cancer research in Malawi (Table 1).

**Table 1:** List of African MEPI Institutions and their US partner institutions.

African MEPI Institutions	Partner institution/s	Programmatic award	Linked/Pilot award

Addis Ababa University, Ethiopia (AU)	University of California, San Diego, Emory University, Johns Hopkins University, University of Wisconsin, University of Alabama at Birmingham	Ethiopia’s Medical Education Consortium for Quality Medical Education & Retention.	None
African Centre for global Health and Social transformation, (ACHEST) Uganda	George Washington University	Coordinating Centre	Not Applicable
University of Botswana, Botswana (UB)	University of Pennsylvania Harvard School of Public Health	Creating Sustainable Medical Education & Health Research Capacity in Botswana.	None
Universidade Eduardo Mondlane (UEM), Mozambique	University of California, San, Diego (UCSD)	The Universidade Eduardo Mondlane/UCSD Medical Education Partnership	UEM-UCSD Surgery Partnership
University of Ibadan, Nigeria	Northwestern University Harvard School of Public Health	Medical Education Partnership Initiative in Nigeria (MEPIN)	None
Kilimanjaro Christian Medical University College, (KCMC) Tanzania	Duke University	KCMC-Duke Medical Education Partnership Initiative.	None
Kwame Nkrumah University of Science and Technology (KNUST), Ghana	University of Michigan	Not Applicable	Ghana Emergency Medicine Collaborative Training Program.
University of Kwa-Zulu Natal (UKZN), South Africa	University of Denver Columbia University University of Colorado University of California, San Diego University of Missouri University of Wisconsin	Enhancing Training, Research Capacity and Expertise in HIV Care (ENTREE).	None
University of Malawi, Malawi	University of North Carolina John Hopkins University	Not Applicable	HIV-associated Malignancies in Malawi.
Makerere University College of Health Sciences (MakCHS), Uganda	John Hopkins University Case Western Reserve University	Medical Education for Equitable Services to All Ugandans (MESAU).	Building Capacity for Cardiovascular Diseases (CVD) Research and Training in Uganda
University of Nairobi, Kenya	University of Washington University of Maryland, Baltimore	Partnership for Innovative Medical Education in Kenya (PRIME-K)	Strengthening Maternal, Newborn & Child Health (MNCH) Research Training in Kenya
Stellenbosch University, South Africa	Johns Hopkins University Morehouse University	Stellenbosch University Rural Medical Education Partnership.	None
University of Zambia, Zambia	Vanderbilt University University of North Carolina University of Alabama, Birmingham University of Maryland, Baltimore	Expanding Innovative Multidisciplinary Medical Education in Zambia.	Improving Maternal and Child Health through Specialty Training in Zambia
University of Zimbabwe	University of Colorado, Denver Stanford University University of Cape Town University College London King’s College London	Novel Education Clinical Trainees and Researchers (NECTAR) program	Improving Mental Health Education and Research Capacity in Zimbabwe.(IMHERZ) Cerebrovascular, Heart Failure, Rheumatic Heart Disease Interventions Strategy Initiative.(CHRIS)

This paper presents an overview of the transformation that MEPI inspired and highlights some of the accomplishments achieved under the programme. This article reports on data compiled from four annual surveys, site visit reports, summary reports from the schools, publications, Technical Working Group (TWG) reports and teleconference reports among the writing team. It was an exciting five-year experience that witnessed African-led innovations in medical education and research, and the power of partnerships. The impact on health systems is too early to measure and will be reported in future.

## MEPI innovations in leadership and partnerships

A unique innovation of MEPI is that unlike some other donor grants, it was awarded directly to the African institution under the principal investigator (PI). The African PIs determined the direction of their programmes within the context of the Request for Application (RFA).

Another innovation is that policy and programme leadership and oversight of MEPI was led by the Principal Investigators Council (PIC), comprising PIs from each grantee institution and representatives from the NIH, HRSA the Office of the U.S. Global AIDS Coordinator (OGAC), and the CC. The PIC elected its chair and vice-chair on an annual rotating basis from one of the PIs. It served as the steering committee for MEPI and met two times each year at a member school. Administrative and financial management in each school was led by the PI coordinating in-country and international partners for programme implementation. An illustration of the strength of African leadership in MEPI is that the PIC emerged during the first year of implementation, as a response to an identified leadership gap. The emergence of the PIC is a significant achievement of MEPI. This group has the potential and capacity to drive and sustain transformation in medical education in Africa and to play the role of credible partners with the international community. The PIC has expanded to engage new health professional cadres and launched a new pan-African multidisciplinary forum named the African Forum for Research and Education in Health (AFREHealth) during the MEPI/NEPI (Nursing Education Partnership Initiative) Symposium in August 2016. This new body is poised to provide leadership in health professions education and research capacity building on the continent.

A welcome outcome of MEPI was the expansion of the activities of the programme to institutions beyond the original grantees. While only 13 schools received grants, representatives from more than 40 medical institutions and other education leaders attended the first MEPI symposium in Johannesburg, South Africa, in 2011, with participation increasing annually. This opened the programme to other schools with the aim of widely spreading MEPI improvements. This was an outcome that was only possible through the foresight of the PIC and demonstrated willingness to share these grants with peers, many of whom might have been viewed as competitors. MEPI was further strengthened by the representation of ministries of health and education of recipient countries during its annual meetings. MEPI now encompasses 60 African schools, representing 37.5% of the over 160 schools of medicine in sub-Saharan Africa.

Site visits for peer learning was another transformative innovation. Grantee institutions were visited for two to three days by education leaders in Africa and from the USA who were faculty members of ACHEST and GWU, PIs from MEPI grantee schools, and programme officers from the NIH or the HRSA. Schools with programmatic awards were visited annually and schools with pilot linked awards every other year. These visits provided opportunities for comprehensive review of the progress, and face-to-face exchanges with stakeholders at the schools. The stakeholders included university vice-chancellors, students, and government officials from health and education ministries. Visiting teams also provided peer technical support and motivational talks and lectures on relevant topics. Adjustments in the programmes were discussed, and the visits were a learning opportunity for visiting PIs and the CC faculty. Individual reports prepared by the MEPI schools together with the site visit reports prepared by the CC were used as key data sources for learning and monitoring performance.

The annual MEPI symposia hosted by one of the schools and organised by the CC provided an opportunity for information sharing, joint learning, and networking across continents. The MEPI community, partners, and international medical education groups converged for annual three-day meetings with up to 350 participants as the largest annual medical education event in Africa. Guest speakers came from Africa, the Americas, Europe, and Asia, and schools showcased their work through oral and poster presentations. The MEPI website was another platform for information exchange and dissemination that served the MEPI community and medical educators around the world. There were also active thematic TWGs as communities of practice, monthly newsletters, webinars, and special-purpose workshops. Over 376 peer-reviewed publications, including a special supplement in the journal *Academic Medicine* (2014), communicated the rich research findings and medical education related accomplishments of the MEPI programme.

Partnerships were a major strength of MEPI, which created a community comprising 60 medical schools in sub-Saharan Africa and 26 in America and Europe. These partnerships facilitated the implementation of MEPI. The engagement of multiple stakeholders within the countries especially the vice-chancellors, professional associations and councils, the ministries of health, education, and finance aligned MEPI with national priorities and ensured sustainability. A network of new medical schools known as Consortium of New Southern African Medical Schools (CONSAMS), and international organisations also participated in this partnership.

These South-South and South-North partnerships enabled the sharing of best practices and collaboration in teaching and research. Examples include visiting professors to the University of Zimbabwe College of Health Sciences (UZCHS) from the USA and Europe, teleconferencing between the Kilimanjaro Christian Medical College (KCMC) and Duke University, virtual teaching between Medical Education for Equitable Services to All in Uganda (MESAU) and John Hopkins University. Other examples include the Kwame Nkrumah University of Science and Technology (KNUST) partnering with the University of KwaZulu-Natal in Emergency Medicine and Stellenbosch University partnering with the UNZA, Kenyatta University, Kenya, the University of Botswana, and Makerere University in the development of Family Medicine [14,15].

The CC faculty brought additional resources and valuable experience, including lessons from Walter Sisulu University in South Africa on local student recruitment, the University of Gezira in Sudan on linking medical education to health systems, the Francophone Africa education models from Mali, and the links with African health systems governance by ACHEST. The faculty from GWU contributed USA medical education perspectives.

In 2014, students established the MEPI Students Network to promote collaboration among MEPI students with ACHEST providing the secretariat. This provided platforms for student dialogue with faculty and university authorities in addressing students’ needs regarding matters such as housing, curricula, library facilities, and the educational environment and potential to improve the quality of education in a number of schools [14].

## MEPI innovations in teaching and learning

MEPI has catalysed positive changes within participating schools most of which had been negatively impacted by economic and political upheavals in their countries. Site visit reports indicate that over the five-year period, the morale in the schools has transformed from one of despondency to optimism. One PI stated that “MEPI has breathed a new life into this school”. At the beginning of MEPI, 80% of the students at the University of Zimbabwe College of Health Sciences (UNZCHS), by a show of hands, indicated that they had no intention of remaining to work in the country, by the end of MEPI, in a similar informal poll, 82% of undergraduate students indicated that they were willing to stay and work in Zimbabwe [14]. This is promising as well as the fact that African governments contributed funds to MEPI and sustained MEPI initiatives testifies to the probability of sustainability of MEPI interventions.

Joint learning was recognised as critical to success and under the guidance of the PIC and the CC, eight TWGs were established and designated as Communities of Practice, [16] namely eLearning, Community Based Education, Competency Based Medical Education, Monitoring and Evaluation, Graduate Tracking, Library and Information Sciences, Medical Education Research and Research Support Centers. Each TWG comprised members from MEPI schools, and the TWG secretariats moved over time from the CC to the schools. The TWGs have remained active through virtual as well face-to-face meetings and conduct special sessions during the annual symposia.

**Figure 1:**
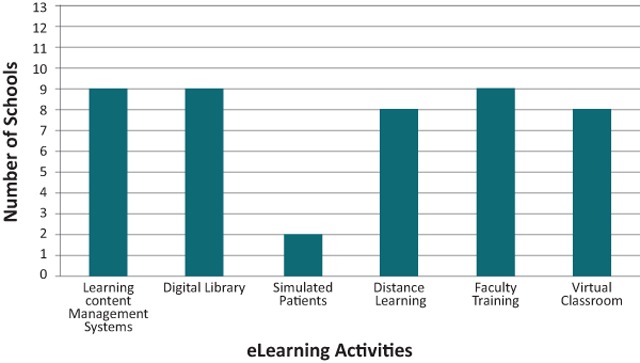
MEPI Supported eLearning Activities (2011–2015). Source: Cumulative data from MEPI Annual Survey, Year 2, 2012; MEPI Annual Survey, Year = 3, 2013; MEPI Annual Survey, Year 5, 2015. Note: Includes date from 13 schools.

All schools embraced e-Learning by enhancing infrastructure, improving internet connectivity, installing more computers, linking off-campus satellite training sites to the internet, restructuring library spaces to facilitate e-Learning, replacing paper-based learning materials, distributing electronic textbooks and tablets, providing electronic subscriptions to journals, adopting use of e-Granary, establishing learning management information systems, podcasting lectures, and establishing bring-your-own-devices systems. Over 5000 tablets were distributed to students with more than 100 textbooks, national health guidelines and teaching materials loaded in memory cards at the Addis Ababa University [17]. Makerere University College of Health Sciences, the UEM, the University of Malawi, and the KCMC now use real-time video links for education interactions with partner schools abroad (Figure 1).

According to the medical students, MEPI support spurred “significant improvement” in their training. “It’s an understatement to say that information technology has revolutionized our learning,” they wrote. “The unprecedented access to new portals of knowledge allows us to take advantage of e-textbooks that we can read any time. We also have new possibilities of interacting with other students and professionals from around the world” [18].

Curriculum reviews were undertaken by all the schools, and new curricula with content enhancement and innovative delivery methods were developed, providing more relevant training and addressing faculty shortages. Seven universities revised curricula to competency-based embracing standards in critical thinking, information management, communication skills, clinical skills, and population health, scientific foundations for medical education, and professional values and attitudes. The establishment of medical education units in nine schools served as the driving force for institutionalising faculty development, teaching and learning innovations, curriculum reform, evaluation of educational interventions, and ensuring compliance with local and international regulatory agencies.

Faculty development was undertaken by providing pedagogy training at home and abroad, research support, continuing professional development, and clinical skills enhancement. For example, at the UZCHS, skills in insertion of cardiac pacemakers and bronchoscopy training abroad and at home incentivised new faculty members.

**Figure 2:**
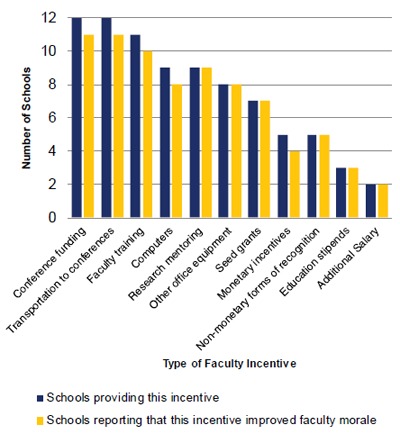
MEPI Supported Faculty Incentives (2011–2015). Source: MEPI Annual Survey, Year 5, 2015. Note: Includes data from 12 schools.

Retention of graduates was a core objective of MEPI. Targeted selection of trainees, support for research activities, advanced training opportunities, and increased access to technology for improving the quality and efficiency of teaching were employed as strategies to retain graduates. Six schools used MEPI funding to hire 51 new staff for a variety of positions, and only four of them were not expected to continue beyond the grant period. School faculty cited the opportunity to attend conferences and present their research as one of the most valued incentives (Figure 2).

**Figure 3:**
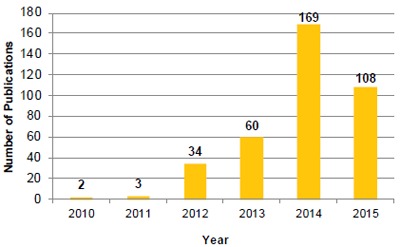
Number of MEPI Publications by year (2010–2015). Source: Data compiled from MEPI Final Year Survey (2015), 13 school level reports, and CC report. Note: Includes data from 13 schools and CC. Data was captured mid-2015.

Building capacity for locally relevant research, a key objective of MEPI, was achieved by funding mentored scholars’ research and establishing Research Support Centres (RSCs) [19]. RSCs facilitated training in research methodology, grant writing, scientific writing, and research administration that contributed to building a sustainable research environment. This improved the schools’ ability to attract funding, recruit and retain faculty, and contribute to the national research agenda and the sustainability of the programmes (Figure 3).

The educational innovations described, and the positive climate created by MEPI, facilitated an increase in the number of undergraduate and postgraduate students enrolled and an improvement in the quality of teaching and learning in all the schools. MEPI contributed to the establishment of one new school in Uganda, Zimbabwe, and Mozambique, two new schools in Zambia, and three in Ethiopia, thus increasing the students enrolled. Increased enrolment of postgraduate students was achieved by improving the teaching and research environment, offering them faculty positions, and targeting specific areas of need such as Emergency Medicine in Ghana, Family Medicine in Ethiopia, Pathology in Malawi and basic sciences teachers training in Zambia. At the UZCHS and the UEM, enrolment in Internal Medicine increased by 200%, and the graduation pass rate at the UZCHS improved by 20% (Table 2).

**Table 2:** Retention of faculty and graduates under the MEPI programme.

School/Universities	Number of Faculty		Graduates		Postgraduates Graduated	

	2010	2015	2010	2015	2010	2015

University of Botswana, Botswana (UB)	50	64	0	80	0	4
Universidade Eduardo Mondlane (UEM), Mozambique	147	194	108	127	12	64
Kwame Nkrumah University of Science and Technology (KNUST), Ghana	3 (emergency physicians)	3 (emergency physicians)	0	129 (emergency nurses)	0	21 (emergency physicians)
*Medical Education for Services to All Ugandans (MESAU), Uganda	490	788	329	598	86	209

* MESAU stands for Medical Education for Equitable Services to All Ugandans. It is a consortium of universities that include Makerere, Mbarara, Gulu, Kampala International University and Busitema University.

**Figure 4:**
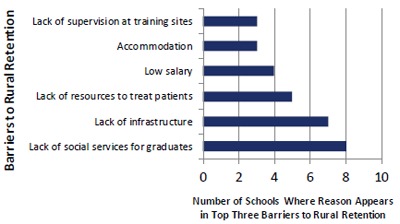
Top Barriers to Rural Retention. Source: MEPI Final Year Survey (2015). Note: Includes data from the 10 schools that reported difficulties with rural retention.

MEPI stimulated growth in community-based and decentralised training [20]. Achieving rural retention of health workers was strengthened by such training, a goal that required close collaboration with the leadership of national governments. Regular meetings with government representatives in the ministries of health led to increased awareness of WHO-documented deterrents to rural retention such as poor practice facilities, lack of supervision, poor housing, poor education for children and low pay. Other innovations that promoted graduate retention included revised admission policies such as preadmission interviews at the UZCHS and affirmative action for defined disadvantaged population groups in Stellenbosch (Figure 4).

**Figure 5:**
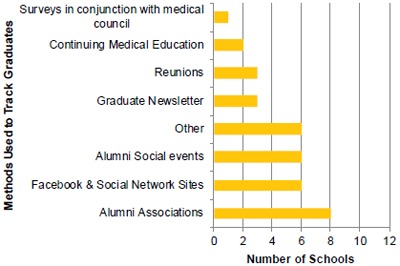
Methods Schools Used to Track Graduates. Source: MEPI Final Year Survey (2015). Note: Includes data from 12 schools.

All schools participated in the Graduate Tracking TWG to assess and monitor retention and distribution of their graduates [21]. Strategies to track graduates included social media, alumni associations and newsletters. National registration councils were also encouraged to set up health workforce information systems in collaboration with training institutions (Figure 5). Despite this effort only four schools had graduate tracking systems in place by the end of the five years; this could indicate that this is not an easy process to establish.

**Figure 6:**
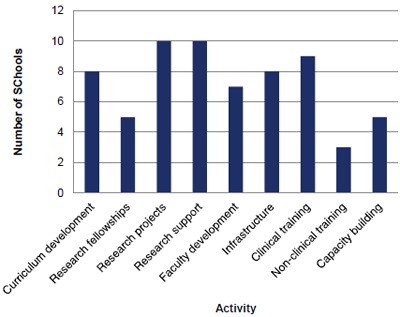
MEPI – Supported Activities Related to HIV/AIDS Care, Treatment, and Prevention (2011–2015). Source: Data from MEPI Final Year Survey (2015). Note: Includes data from 12 schools.

To improve HIV/AIDS service delivery, schools revised curricula to incorporate new HIV/AIDS guidelines, evidence-based treatments, care management, and diagnostics. In addition, schools focused on improving pre-service training on HIV/AIDS-related topics for nurses, midwives, technicians, physician assistants, and other non-physicians on providing in-service training for physicians on co-infection management, prevention, and medication access and adherence (Figure 6).

Sustainability was addressed by aligning interventions with national priorities, recruiting support from university leadership, ensuring access to national budgets, establishing self-sustaining structures such as RSCs and medical education units. MEPI government support in Zimbabwe was exemplary where the Ministry of Education provided a matching grant to MEPI and re-opened a closed medical school. The Permanent Secretary travelled to address the second symposium in Ethiopia. MEPI achievements are summarised in Table 3.

**Table 3:** MEPI Schools’ Achievements Summary.

Capacity building	Number of Number of schools reporting Achievement

Augmented faculty training and continuing medical education (CME) activities	7 schools
Curriculum review/reform underway	9 schools
New teaching methodologies	7 schools
Improved medical education infrastructure (teaching resource centers, medical education units and departments of medical education)	7 schools
E-learning programs in place	9 schools
Increased Clinical specialty/residency training Programs	6 schools
MEPI supported physical infrastructure renovation (renovation of classrooms, laboratories, skills labs, rural sites etc.)	7 schools
MEPI supported digital infrastructure renovation (improving internet connectivity and access to computing devices etc.)	8 schools
Investments in skills labs	5 schools
**Retention**	

Incentives provided to faculty through MEPI	9 schools
Faculty hired with MEPI funds	4 schools
Mechanism to recruit graduates as junior faculty	2 schools
Graduate tracking systems in place	4 schools
Community-based/rural training	7 schools
**Research**	

Development of research support centers	9 schools
Research Training for Students	10 schools
Research Training for Faculty	10 schools
Access to new research funding opportunities as a result of MEPI enhancement of research capacity	5 schools
Research mentoring program for students	8 schools
**Sustainability**	

Commitment of monetary and non-monetary support from government and non-government sources to continue MEPI programs	10 schools
Future Plans in place to promote sustainability of MEPI programs	10 schools
**Communities of practice**	

South to south consortia/partnerships	10 schools
South to North consortia/partnerships	9 schools
Technical Working Group participation	10 schools

Over the course of MEPI challenges were inevitably encountered, the five year period was not long enough to turn some of the innovative ideas into culture, scarcity of experienced researchers and faculty remained an obstacle to advancement of some of the innovations and their sustainability. Faculty recruitment and retention remained a big obstacle. ICT was found to be a powerful tool in medical education but slow internet connectivity, inadequate planning and training still caused limitations to its full implementation.

## Discussion and looking to the future

MEPI is the biggest intervention in scaling up the education and training of health workers in Africa while the MEPI symposia convene the largest number of multidisciplinary health professions educators. MEPI funding has ignited enthusiasm that resulted in new medical schools, rural training sites, and matching funds. It increased the quality and quantity of health care workers. The majority of these expressed commitment to remain in-country. In addition, it caused a culture change in how medical schools collaborate instead of competing with each other and encouraged them to form networks to share best practice and leverage resources. Another dramatic shift has been the adoption of information communication technologies to enhance teaching, expand student and faculty access to electronic education materials and current scientific literature. MEPI has reviewed curricula to make them responsive to the population health needs.

The MEPI work complements the recommendations made by the High-Level Commission on Health Employment and Economic Growth to stimulate and guide the creation of at least 40 million new jobs in the health and social sectors, and to reduce the projected shortfall of 18 million health workers, primarily in low- and lower-middle-income countries, by 2030 [12]. It is hoped that MEPI has and continues to add to the number of health workers trained in Sub-Saharan Africa.

Scaling up the health professions education should take into account the importance of an adequate skills mix and recognition of the other carders. This is important and it is one of the major thrusts taken up by the African Forum for Research and Education in health (AFREhealth) which is a new multidisciplinary organisation that has been formed out of MEPI and NEPI. This new organisation is committed to developing health professionals’ education and research in Africa, sharing best practices, and reducing health disparities building on the MEPI work [22].

Sustaining MEPI achievements will be determined by the leadership provided by Africans, continued stability and economic growth in African countries. These conditions are needed for creating the fiscal space to employ and retain high-calibre faculty and graduates, supporting research, and maintaining the educational infrastructure. The international community has vested interest in that MEPI has demonstrated the availability of opportunities for collaboration and their evident desire to improve performance of African partners. Further, high-quality African health professionals represent a global public good needed for ensuring global health security and achieving global health goals. While five years is a short time in medical education and health system development, meaningful partnerships and the promising innovations demonstrated by MEPI have sparked a medical education revolution that will lead to better health outcomes for Africa and the world.
